# Analysis of the genetic diversity and population structure of *Monochasma savatieri* Franch. ex Maxim using novel EST-SSR markers

**DOI:** 10.1186/s12864-022-08832-x

**Published:** 2022-08-16

**Authors:** Wanling Yang, Zhiyi Bai, Fuqiang Wang, Mingzhu Zou, Xinru Wang, Jiankun Xie, Fantao Zhang

**Affiliations:** 1 College of Life Sciences, Jiangxi Normal University, Nanchang, 330022 China; 2 Yichun Academy of Sciences, Yichun, 336000 China

**Keywords:** EST-SSR, Genetic diversity, Wild germplasm resources, *Monochasma savatieri*, Population structure

## Abstract

**Background:**

*Monochasma savatieri* Franch. ex Maxim is a medicinally valuable herb. However, the collection and protection of the wild germplasm resources of *M. savatieri* are still insufficient, and their genetic diversity and population structure have been poorly studied.

**Results:**

We collected and examined 46 *M. savatieri* individuals from Fujian, Hunan, Jiangxi, and Zhejiang provinces for genetic diversity and population structure, using 33 newly developed expressed sequence tag-simple sequence repeat (EST-SSR) markers. Applying these markers, we detected a total of 208 alleles, with an average of 6.303 alleles per locus. The polymorphic information content varied from 0.138 to 0.884 (average: 0.668), indicating a high level of polymorphism. At the population level, there was a low degree of genetic diversity among populations (I = 0.535, He = 0.342), with Zhejiang individuals showing the highest genetic diversity among the four populations (Fst = 0.497), which indicated little gene flow within the *M. savatieri* populations (Nm = 0.253). Mantel test analysis revealed a significant positive correlation between geographical and genetic distance among populations (*R*^2^ = 0.3304, *p* < 0.05), and structure and principal coordinate analyses supported classification of populations into three clusters, which was consistent with the findings of cluster analysis.

**Conclusions:**

As a rare medicinal plants, the protection of *M. savatieri* does not look optimistic, and accordingly, protective efforts should be beefed up on the natural wild populations. This study provided novel tools and insights for designing effective collection and conservation strategies for *M. savatieri*.

**Supplementary Information:**

The online version contains supplementary material available at 10.1186/s12864-022-08832-x.

## Background

*Monochasma savatieri* Franch. ex Maxim, a perennial medicinal plant in the Scrophulariaceae family, is endemic to China and Japan, where it grows preferentially on sandy hillsides and in grassy clusters. In China, it is mainly distributed in southeastern regions, including the provinces of Jiangxi, Fujian, Hunan, and Zhejiang [1, 2]. The whole plant of *M. savatieri* can be used for medicinal purposes, including the treatment of colds, cough, pneumonia, fever, toothache, and irregular menstruation [3–5]. Preparations of the plant are characterized by a wide range of biological activities, including antioxidant, antibacterial, and antiviral activities, which are assumed to be attributed the main medicinally effective components, namely, phenylpropanoids, flavonoids, alkaloids, saponins, and polysaccharides [6, 7].

*M. savatieri* has a long history of medicinal use and is one of the essential component of Yanning granules, which has been designated a “national protected traditional Chinese medicine variety” [8, 9]. However, as an important and valuable medicinal plant, the artificial cultivation of *M. savatieri* has been unsuccessful. In recent years, there has been an increase in the market demand for *M. savatieri*, which has often led to limitations in the supply of its wild resources. Such scarcities among wild populations can be attributed to the fact that *M. savatieri* is a root hemiparasite that has stringent habitat requirements and a naturally weak reproductive capacity [10]. Moreover, the seeds *M. savatieri* are characterized by a short period of dormancy, and germination rates decline sharply with a prolongation of storage time. Consequently, if, on reaching maturity, the seeds lack suitable germination conditions, they will rapidly lose vitality [11]. Owing to a combination of unfavorable factors, including over-harvesting and habitat destruction, the natural habitats of wild resources of *M. savatieri* have sharply deteriorated and reduced in size. Their number of individuals and size of natural population have markedly declined in recent years owing to the short seed dormancy and inherently poor regeneration. This has resulted in substantial losses of genetic resources, as indicated by a survey conducted in China by Chen et al. [12], who found that wild resources of *M. savatieri* in almost all investigated regions showed sharp declines. Moreover, in Japan, *M. savatieri* has long been listed as an endangered species [13]. Consequently, conservation of the wild resources of *M. savatieri* is considered a matter of particular urgency. Currently, however, there is a notable lack of information regarding the genetic variation of *M. savatieri*, which thereby hinders evaluations of the genetic status of this species and makes it difficult to formulate appropriate scientifically based resource conservation strategies.

Analyses of species genetic diversity and population structure play key roles in genetic resource conservation and plant breeding [14, 15]. In this regard, a range of molecular markers, including random amplified polymorphic DNA (RAPD), amplified fragment length polymorphism (AFLP), sequence-related amplified polymorphism (SRAP), simple sequence repeat (SSR), and single-nucleotide polymorphism (SNP) markers, have been widely used to assess the genetic diversity of species and reveal their population structures [16–21]. Among these, SSRs are widely regarded as ideal molecular markers, owing to their significant advantages of co-dominance, wide distribution, high mutation rate, high polymorphism, necessity for only small quantities of template DNA, and relatively low cost [22, 23]. Depending on the original sequence source of SSR loci, these markers can be divided into genomic SSR markers and expressed sequence tag (EST) SSR markers [24]. Compared with genomic SSRs, the use of EST-SSRs is economical, highly transferable among plant species, and less susceptible to invalid alleles [25, 26]. Moreover, EST-SSRs are derived from the expressed regions of genes, and consequently polymorphisms are more likely to be directly associated with gene function, thereby making it easier to identify and characterize certain key traits [27]. The use of these markers is thus considered particularly beneficial with respect to plant genetic mapping, resource identification, genetic diversity evaluation, and population structure analyses [28–30]. To date, studies on the genetic diversity of *M. savatieri* are still limited, and one of the most important reasons is the lack of effective molecular markers for the species.

In this study, great efforts have been made to collect and preserve the wild germplasm resources of *M. savatieri* from the major distribution provinces of China. Meanwhile, we detected an abundance of EST-SSRs in the assembled transcripts of the full-length transcriptome data of *M. savatieri*, and from among these, we developed reproducible polymorphic EST-SSR markers to assess the level of genetic diversity and genetic structure for the collected wild germplasm resources of *M. savatieri*. The collected wild germplasm resources and genetic information will provide novel insights for the conservation, utilization and breeding strategies development of *M. savatieri*.

## Results

### Characterization of EST-SSR loci

**Fig. 1:**
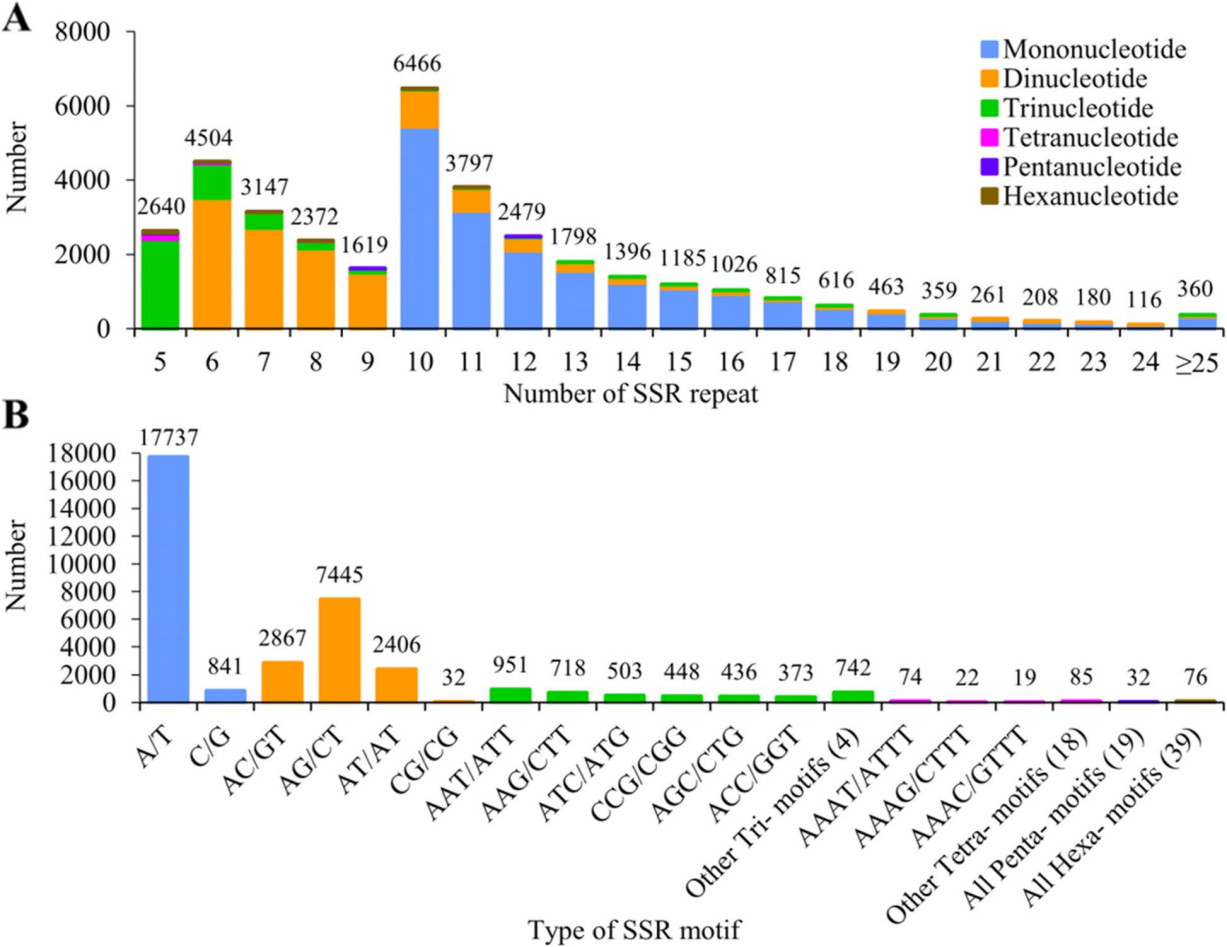
Characterization of SSR loci. **A** Distribution of SSR repeat number in *Monochasma savatieri*. **B** Distribution of SSR motif types in *Monochasma savatieri*

In total, we detected 35,807 EST-SSR loci distributed among 18,279 of a total of 40,970 transcript sequences (98.98 Mb) (The data were deposited and are available at https://github.com/AWan222/LRCyc). The frequency of SSR distribution (number of SSR/total transcripts) was 87.40%, with an average of one locus per 2.76 kb. In addition, we found that 9078 sequences contained more than one SSR loci, and that there were 6363 (17.77%) compound SSRs. The SSR types were abundant, ranging from mononucleotide to hexanucleotide repeats, and the number of each type varied. Among these, mononucleotide (18,578, 51.88%) and dinucleotide (12,750, 35.61%) EST-SSRs were the most abundant repeat types, followed by trinucleotide loci (4171, 11.65%), with less than 1.00% of the total being represented by tetra-, penta-, and hexa-nucleotides (Table 1). The number of repeats varied greatly among the different repeat types of SSRs. The highest frequency of SSR repeat types occurred with 10 repeats, with 6466, accounting for 18.06%, followed by 6 (4504, 12.58%) and 11 (3797, 10.60%) repeats (Fig. 1). The repeats of mononucleotide SSRs were mainly concentrated in the range of 10–20 times, and the number of other SSR repeats were mainly concentrated in the range of 5–10 times. Among the identified SSRs, a total of 95 types of motif were detected (Fig. 1). Tetra- and hexa-nucleotide repeat types exhibited the highest number of motif types (21 and 39 types, respectively). Moreover, among different repeat motif types, the A/T repeat motif was the most abundant (17,737, 49.54%) followed by AG/CT (7445, 20.79%), AC/GT (2867, 8.01%), AT/AT (2406, 6.72%), and AAT/ATT (951, 2.66%). These findings provide a solid foundation for the development of EST-SSR markers in *M. savatieri*.

**Table 1:** Occurrence of SSRs in the transcripts of the *Monochasma savatieri*

Type of SSR	Number	Proportion in all SSRs (%)	Distribution frequency (%)	Mean distance (kb)	Length (bp)
Mononucleotide	18,578	51.88	45.35	5.33	10–124
Dinucleotide	12,750	35.61	31.12	7.76	12–68
Trinucleotide	4171	11.65	10.18	23.73	15–105
Tetranucleotide	200	0.56	0.49	494.89	20–44
Pentanucleotide	32	0.09	0.08	3093.12	25–60
Hexanucleotide	76	0.21	0.19	1302.37	30–66
Total	35,807	100	87.40	2.76

### Genetic diversity

**Fig. 2:**
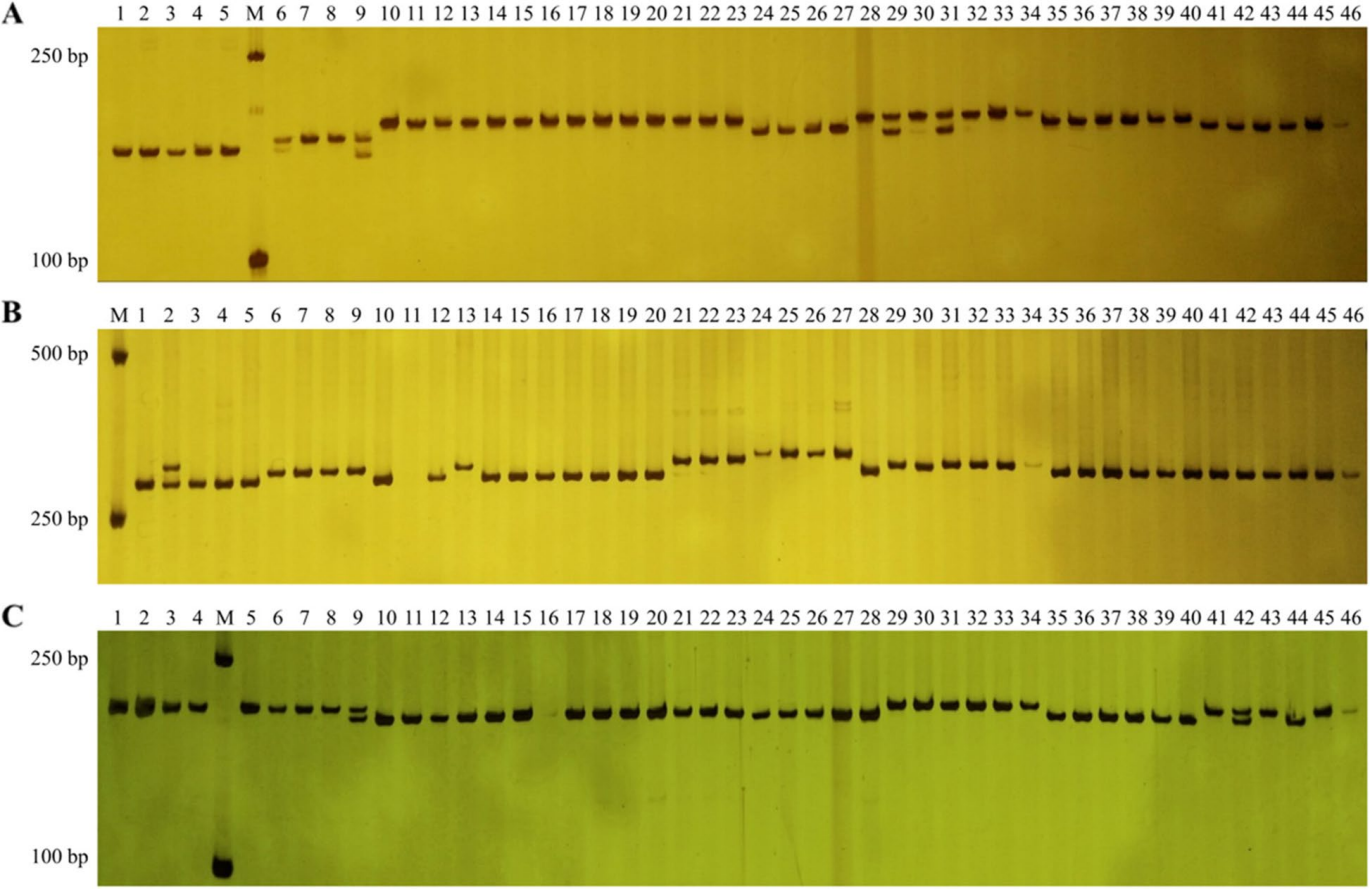
Results of the PCR amplification of 46 *M. Monochasma savatieri* germplasm resources using the EST-SSR markers (**A**) LRC-19013-3, (**B**) LRC-35320-1, and (**C**) LRC-10353-1. M: DNA Ladder; 1 to 46: materials of the 46 *Monochasma savatieri* individuals listed in Supplemental Table 1

For the purposes of EST-SSR primers screening, we randomly selected the DNA of 10 different *M. savatieri* individuals, among which, we developed 33 highly polymorphic SSRs that were subsequently used to assess the genetic diversity and genetic relationships among the 46 *M. savatieri* specimens obtained from different wild habitats in China (Table 2). We accordingly succeeded in amplifying a total of 208 alleles, with the numbers of alleles scored for the 33 loci ranging from 3 (LRC-60747-1) to 11 (LRC-19013-3) with an average of 6.303 (Fig. 2). The average MAF ranged from 0.130 to 0.924 (average: 0.424), whereas the GD ranged from 0.143 to 0.894 (average: 0.706), thereby indicating differences in the polymorphisms of the EST-SSR loci. The PIC ranged from 0.138 to 0.884 (average: 0.668), with the values indicating that 31 SSR loci (93.94%) were highly polymorphic (PIC ≥0.5). Of the remaining two loci, one was moderately polymorphic (0.25 < PIC < 0.5) and the other showed low polymorphism (PIC ≤0.25).

**Table 2:** Genetic diversity parameters of the newly developed 33 EST-SSR markers across the 46 *Monochasma savatieri* individuals

Primer name	Primer sequence (5′-3′)	SSR motif	Na	MAF	GD	PIC
LRC-19-1	F:ATACAGTTCGCCGAGCAATC	(CT)12(CA)7	4.000	0.478	0.606	0.526
	R:TGTGACAGAGCAAGCCAAAC					
LRC-38-2	F:TATTGCGTTCTTTGCTTCCA	(T)17	8.000	0.261	0.817	0.791
	R:CGACCATCACCTCTAGCTCC					
LRC-491-1	F:AGTTAATGATGCCGATTGCC	(GA)10	5.000	0.435	0.704	0.659
	R:TAATCTCCTCGCCAAATTGC					
LRC-955-2	F:AGATGCGGTAAACCACAAGG	(T)10	5.000	0.435	0.721	0.682
	R:AAGAGGGCATTGGCTTTTCT					
LRC-1030-1	F:AAGCTCTGTAATGGCGTTCG	(CT)9	6.000	0.522	0.672	0.640
	R:TGAATATGCGCTTCGATTGT					
LRC-1279-1	F:TAACCAGAATCCACGTGTCG	(T)10	7.000	0.565	0.635	0.605
	R:AAGGTCCCATAAGCATCAGC					
LRC-3853-1	F:CTCGCACACTATCTTCCACG	(GGCA)6	7.000	0.304	0.796	0.767
	R:AGGGATGATGGATACGGATG					
LRC-4136-1	F:TCGCCATAACTCCGTCTTCT	(AG)6	6.000	0.565	0.636	0.606
	R:GCGAAAGCTATGCCGACTAC					
LRC-4412-1	F:TCCAGTTTTCCATCACCACA	(A)20	4.000	0.543	0.609	0.549
	R:GCGTCGTTGTAATCATGGTG					
LRC-6564-1	F:ATTCAGGCTTCAGCAGCAAC	(AG)16	10.000	0.130	0.894	0.884
	R:GTCGTTCAGCAGTGGGATCT					
LRC-8962-2	F:AGAACAAGTCGTCGGAGCAT	(T)21	4.000	0.489	0.619	0.547
	R:TGCCTTCCCTTGACACTACA					
LRC-9052-1	F:AGATCGATGTGCTCAGCCTT	(GC)7	4.000	0.413	0.680	0.621
	R:GTCTCAACAAGCGACGTTCA					
LRC-10353-1	F:CGATCTGCTCACAGAACCAA	(CGG)5	7.000	0.228	0.813	0.786
	R:CAATCGCTGCAGATCGTTTA					
LRC-18371-1	F:TTGACAAGGCATGCAAATTC	(TTC)5 T(TA)14	6.000	0.435	0.693	0.641
	R:TGAGTTCAACCACTTGGGAG					
LRC-18468-1	F:CCACACGCAGTATTTGGTGA	(CT)6	6.000	0.457	0.702	0.660
	R:TGCTCGTAATGTGGCTCAAG					
LRC-18556-1	F:TTGCAGAAGAATTGCATTGG	(G)10	7.000	0.435	0.712	0.671
	R:CACACGCTGCAACTGGTATT					
LRC-19013-3	F:TATTTCACTGAGGTGCGAGC	(ATGT)9	11.000	0.304	0.840	0.823
	R:TAACGACCACCATTAACGCA					
LRC-21021-1	F:TACACACAATGCATGCCTCA	(C)12	8.000	0.283	0.818	0.794
	R:GCATGATCCGAAGAGGAGAA					
LRC-22140-1	F:AGTTTGCCGACTCAGAAGGA	(G)14	7.000	0.500	0.656	0.606
	R:CCTTTGCAGATTTAGAGCCG					
LRC-24756-2	F:TCTCTGCATGCATCCACTTC	(A)11	7.000	0.391	0.758	0.726
	R:GGCGACGTAGTCATGGAGTT					
LRC-25132-1	F:AGGCGAGTTGGTTTCAGCTA	(ATT)16	8.000	0.261	0.833	0.812
	R:GAAACATGCGTTTGGTGTTG					
LRC-28075-1	F:CTTTCGCCGTTCAAGTTTTC	(T)13	6.000	0.391	0.753	0.719
	R:CTCGTTCTTGAGCATGTGGA					
LRC-34475-2	F:TTGGTGCACCTGCTATGTTT	(AT)18	7.000	0.283	0.796	0.766
	R:CAGAGTGAAACCACAGCCAA					
LRC-35320-1	F:GATTGGCCTCACTTGGTCTC	(AAATT)5	8.000	0.283	0.823	0.800
	R:TTGCAATGCCATGAACAAAC					
LRC-36979-3	F:TCCATGATTGAAGCTTCTCG	(TG)15	10.000	0.283	0.852	0.837
	R:TGCAACAAGGAAGAGCAATG					
LRC-43046-1	F:ATTCTCCGTTGGGAGGTTCT	(TA)7	8.000	0.435	0.737	0.706
	R:AGGAACCAGCTGCACAATCT					
LRC-50242-1	F:TGGAGATTACTTCGCAGCCT	(GA)11	4.000	0.457	0.651	0.584
	R:CCGATGGTGATTATTGGACC					
LRC-55216-1	F:CTGCAAGTGACATGATGGCT	(CAT)5	4.000	0.652	0.519	0.470
	R:GCGGAATTGACTGTGAACCT					
LRC-55844-1	F:ATACTCGTTCACCCAATCGC	(T)10C(CT)9	6.000	0.446	0.729	0.697
	R:TAGCTCCACGATGAAAGGCT					
LRC-60747-1	F:GAAGTGGTGGCACTGAAGGT	(TA)22	3.000	0.924	0.143	0.138
	R:GCCACTGCCTCTTCTCAGAC					
LRC-63796-1	F:CCATTTGCACTCCGGATACT	(ATTG)5	5.000	0.478	0.689	0.647
	R:ATCTCCGCCACTGATGTAGG					
LRC-68008-1	F:GGAAACCAACCTGTGCCTTA	(AT)7	6.000	0.457	0.709	0.671
	R:GGTTTACGGCGAAATCTTGA					
LRC-68284-2	F:CGGACTTCCAATTTCGCTTA	(TTTA)5	4.000	0.478	0.668	0.615
	R:CCACATAGCTTTACCGGCAT					
Mean			6.303	0.424	0.706	0.668

The results obtained for population-level indexes of genetic diversity in each population are shown in Table 3. The number of alleles (Na) for each locus at the population level ranged from 1.909 to 2.273 (average: 2.068), whereas the number of effective alleles (Ne) ranged from 1.682 to 1.823 (mean: 1.747). Among the four populations, we recorded the highest values of Na and Ne in the ZJ and JX populations, respectively. Expected heterozygosity (He) at the population level varied from 0.314 to 0.362 (mean: 0.342) and Shannon index (I) values obtained for the four populations ranged from 0.481 to 0.583 (mean 0.535), with both indexes indicating the highest genetic diversity in the ZJ population, whereas the FJ population was characterized by the lowest genetic diversity. Furthermore, analysis of the percentage of polymorphic loci (PPL) revealed that the FJ and ZJ populations had the lowest (63.64%) and highest (75.76%) PPL values respectively, with a mean value among populations of 70.45%. These results accordingly indicated that the ZJ population has the highest genetic diversity, whereas comparatively, the JX, HN, and FJ populations are characterized by relatively lower genetic diversities.

**Table 3:** Analysis of the genetic diversity of four *Monochasma savatieri* populations

Population	N	Na	Ne	I	He	uHe	PPL	F
FJ	8.697	1.939	1.682	0.481	0.314	0.334	63.64%	0.872
HN	10.788	1.909	1.690	0.498	0.331	0.347	69.70%	0.954
JX	7.758	2.152	1.823	0.578	0.361	0.386	72.73%	0.949
ZJ	17.212	2.273	1.793	0.583	0.362	0.373	75.76%	0.949
Mean	11.114	2.068	1.747	0.535	0.342	0.360	70.45%	0.933

**Fig. 3:**
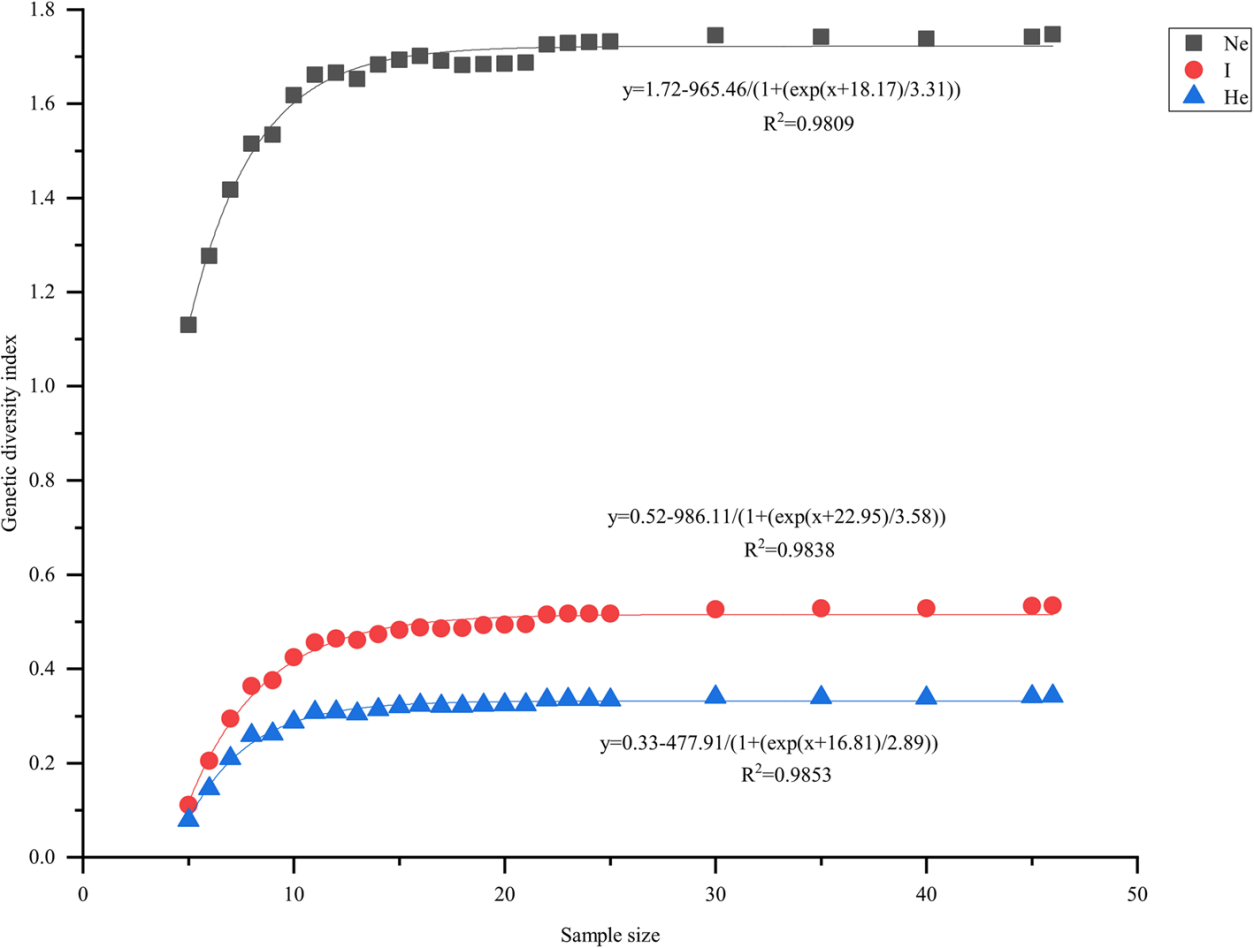
Fitting curves between sample size and different genetic diversity indexes

Sample size is an important factor in the analysis of genetic diversity, because it affects genetic diversity indexes. To verify the accuracy of the genetic diversity results using 46 *M. savatieri* samples, we randomly selected different individuals from 46 samples and constructed 26 groups of different sample sizes with three replicates for each group to plot the trends of the Ne, I and He indexes. As shown in Fig. 3, the genetic diversity indexes Ne, I and He increased with an increase in the effective sample size. However, when the sample size reached approximately 25, the genetic diversity indexes stabilized and no longer increased significantly with increase in sample size. Therefore, the genetic diversity analysis with the 46 *M. savatieri* samples in this study was accurate and effective.

### Genetic differentiation

To gain estimates of genetic differentiation, we performed AMOVA analysis at three levels, namely, among populations, within populations, and within individuals. We accordingly established that 49.74% of the total genetic variation originate from variability within the populations, whereas 48.36% is attributed to differences among populations, and only 1.91% ascribed to within-individual differences (Table 4). F-statistics analysis revealed that the inbreeding coefficient (Fis) and overall fixation index (Fit) were 0.962 and 0.981, respectively, and that the genetic differentiation coefficient (Fst) was 0.497 (Fst > 0.25), indicating a relatively large genetic differentiation among the populations. In addition, we determined a gene flow (Nm) value of 0.253 (Nm < 1), which tends to indicate that the genetic differentiation among population may be caused by migration or genetic drift.

**Table 4:** Analysis of the molecular variance (AMOVA) of the 46 *Monochasma savatieri* individuals

Source	df	SS	MS	Est. Var.	%	*P*-value
Among populations	3.000	492.110	164.037	6.807	49.74	0.001
Within populations	42.000	566.879	13.497	6.618	48.36	0.001
Within individuals	46.000	12.000	0.261	0.261	1.91	0.001

### Population structure

**Fig. 4:**
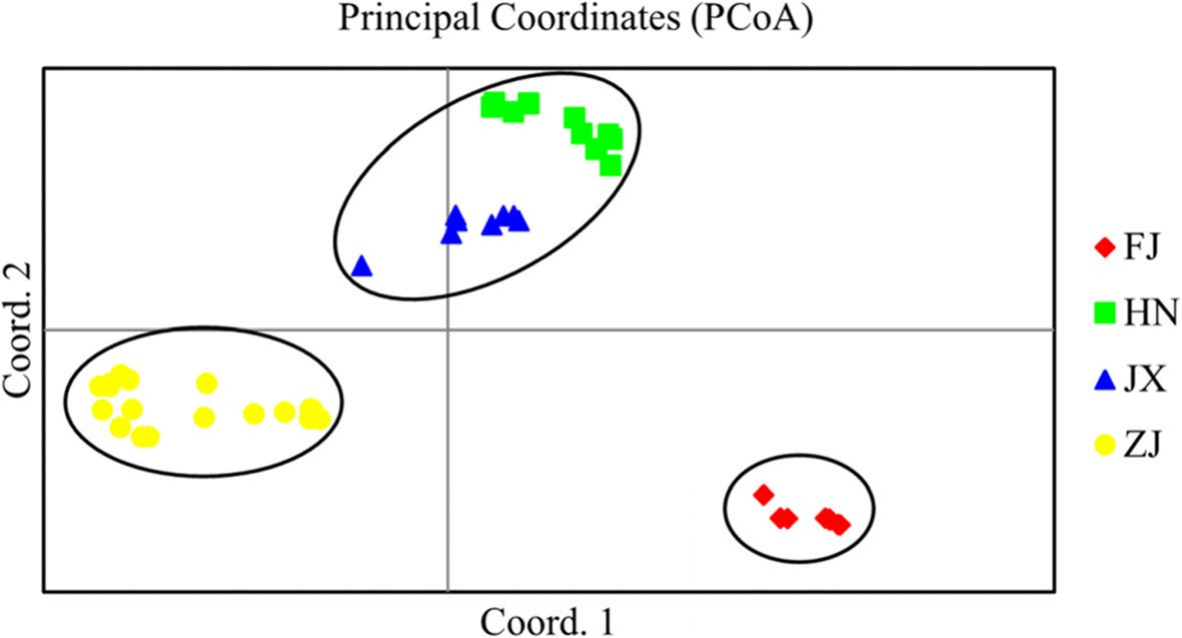
A principal coordinate analysis (PCoA) plot for the four *Monochasma savatieri* populations showing the separation into three main clusters

**Fig. 5:**
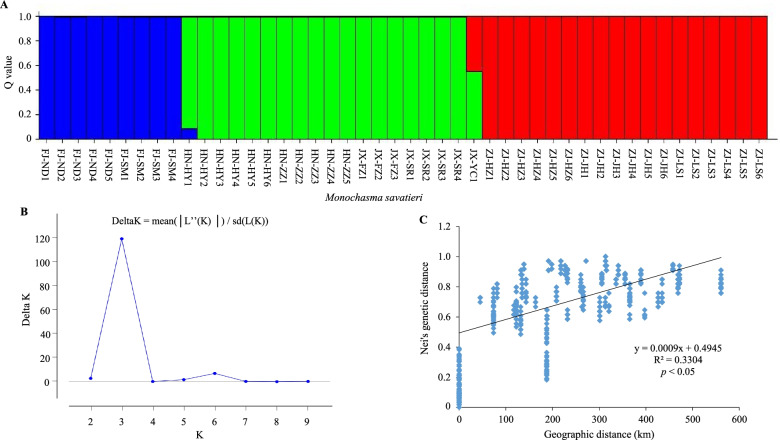
**A** The structure of the *Monochasma savatieri* population inferred based on Bayesian clustering of the EST-SSR markers data. **B** A graph showing the relationship between ΔK and K values. **C** The relationship between the pairwise estimates of genetic distance and the corresponding geographical distances among the four populations of *Monochasma savatieri*

To gain an understanding of the structural and genetic characteristics of the *M. savatieri* populations, we subjected the 46 target individuals to PCoA, UPGMA, and structure analyses. Our PCoA analysis was based on the use of a correlation genetic similarity matrix, showing the first three principal coordinates with Eigen value effects of 20.77, 18.23, and 11.78% respectively, the derived scatter plot of which discriminately divided the 46 individuals into three main groups (Fig. 4). Notably, these three PcoA clusters appeared to be identical to those identified based on structure analysis, representing the natural distribution of *M. savatieri*. Structure clustering analysis revealed that the maximal ΔK value (120.776) was obtained for a K value of 3 (Fig. 5), thereby indicating that the 46 *M. savatieri* individuals could be separated into three distinct genetic clusters (Fig. 5). Specifically, the genetic structure map revealed that the nine individuals in the FJ population grouped in Cluster 1 (blue cluster), the 19 individuals in the HN and JX populations grouped in Cluster 2 (green cluster), and the remaining 18 individuals from the ZJ population were collected in Cluster 3 (red cluster). Consistent with the results of the PCoA analysis, these findings thus indicated that gene penetration among the different *M. savatieri* populations was relatively low.

**Fig. 6:**
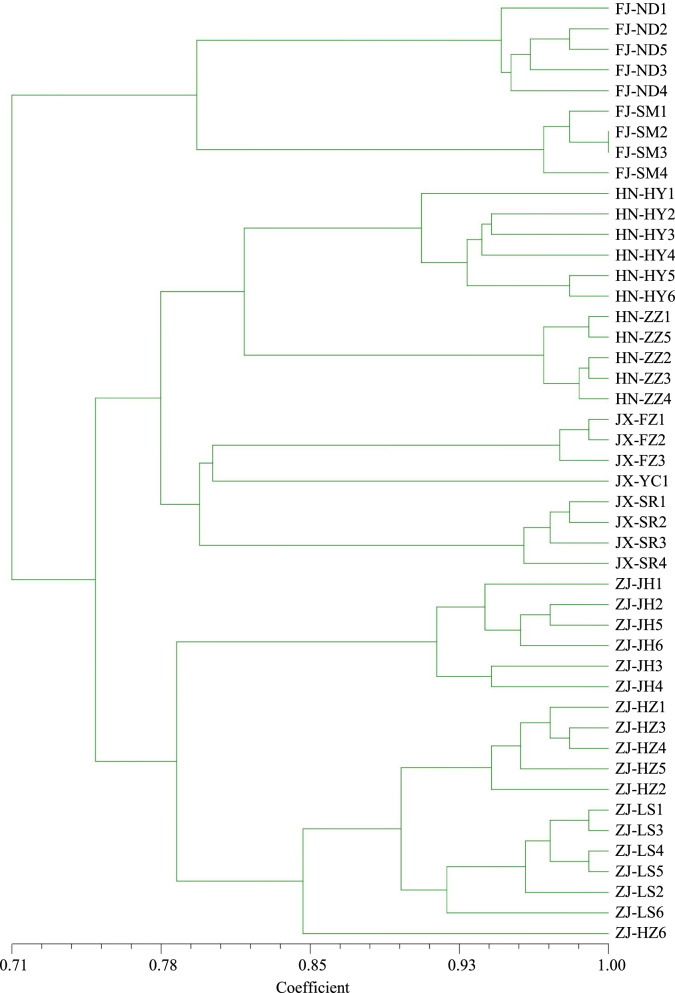
A UPGMA dendrogram of the 46 *Monochasma savatieri* individuals

The same three-way partitioning of populations was similarly confirmed by the findings of UPGMA cluster analysis. The UPGMA dendrogram was constructed based on Nei’s genetic distance and accurately reflects the genetic relationships among and within populations. The tree showed that the 46 *M. savatieri* individuals from the four populations could be divided into two major clusters (Fig. 6), with Cluster 1 comprising exclusively the FJ population and Cluster 2 containing all individuals from the remaining three populations, which were further divided into two short branches, with populations (HN and JX) forming one short branch and the ZJ population comprising the other. Furthermore, both the UPGMA dendrogram and STRUCTURE clustering indicated that the clustering of populations tended to be consistent with the geographical distance among populations. These observations were supported by our Mantel test results, which indicated a significant correlation between genetic and geographical distances (*R*^2^ = 0.3304, *p* < 0.05) (Fig. 5).

## Discussion

*M. savatieri* is an important and rare medicinal plant found only in southeast China and the islands of Kyushu in southwestern Japan [12]. In recent years, as the market demand for *M. savatieri* increased, overharvesting and habitat destruction caused its wild germplasm resources to reduce excessively. Therefore, it is of great importance to collect and analyze the germplasm resources of *M. savatieri* for the formulation of protection strategies and utilization of the wild resources. In this study, great efforts were made to collect and preserve the wild germplasm resources of *M. savatieri* from the major distribution provinces of China. This is the most reported current collection for the wild germplasm resources of *M. savatieri*. The abundant germplasm resources of *M. savatieri* will provide considerable opportunities for future genetic research and breeding application.

Molecular markers play a pivotal role in genetic research and molecular breeding. However, available resources for molecular markers in *M. savatieri* are very scarce. In this study, we developed a novel set of EST-SSR markers and used them to determine the genetic diversity of *M. savatieri* populations. To the best of our knowledge, this is the first study that has sought to assess the genetic diversity of this important medicinal plant species. The frequency of EST-SSRs in *M. savatieri* is comparable to that of found in *Rhododendron fortunei* [31] and *Cocos nucifera* [32], although somewhat lower than that previously reported for *Pongamia pinnata* [33] and *Stephanandra incisa* [34], but notably higher than that in *Paeonia suffruticosa* [35] and *Cephalotaxus oliveri* [36]. These findings accordingly reveal the relatively broad range of SSR densities among plants at the species level. With respect to *M. savatieri*, we identified mono- and dinucleotides as the predominant types of EST-SSR repeat*.* This pattern contrasts to a certain extent with that in a range of other plants, including *Corchorus* spp. [37], *Lycium barbarum* [38], and *Curcuma alismatifolia* [39], for which di- and tri-nucleotide repeats predominate. We speculate that this disparity could be attributable to the relatively long evolutionary history of *M. savatieri* [40]. Furthermore, among the different repeat motifs, we identified A/T (17,737, 49.54%) as the predominant type in *M. savatieri*. Notably, the proportion of A/T motifs was found to be considerably higher than that of G/C motifs, which is consistent with the pattern reported for numerous plants, such as *Opisthopappus* [41], *Pinus koraiensis* [42], and *Phoebe bournei* [43].

Genetic diversity is an important prerequisite for species adaptation to environmental change and the development of disease resistance, the levels of which determine the viability and evolutionary potential of populations, and are often used to predict species trends and potential dangers [44]. The use of molecular markers, such as RAPD, AFLP, and SSR, has been shown to be an accurate and reliable approach for analyzing the genetic diversity of plant populations [45], among which, SSRs are widely employed owing to their strengths. In this study, we used 33 novel developed EST-SSR primers to examine the genetic diversity of *M. savatieri* specimens collected from 10 geographically separated wild areas, the results of which indicated a large variation in the number of alleles of each locus, ranging from 3 to 11 with a mean value of 6.303. The average GD and PIC values determined for the loci, 0.706 and 0.668, respectively, were found to be higher than those reported for some plants, such as those in the genera *Polygonatum* [46] and *Perilla* [47], indicating the high polymorphism of these loci. Additionally, the differences in the values for each locus indicate that these 33 loci are characterized by significant genetic variation among the populations, thereby highlighting their importance with respect to the evaluation of *M. savatieri* germplasm resources. The ploidy level, defined as the number of sets of chromosomes in the nucleus, is a crucial characteristics for studying biodiversity and developing strategies for plant breeding. In this study, within the expected PCR product sizes, the overwhelming majority of alleles of amplification loci of the 33 SSR markers in each germplasm were one or two, whereas three alleles existed only in a few loci. Therefore, it was speculated that *M. savatieri* was a diploid or triploid plant, but additional approaches, including flow cytometry and chromosome counting, were requested to further define the ploidy level of *M. savatieri*.

Analysis of *M. savatieri* genetic diversity at the population level revealed that He and I ranged from 0.314 to 0.362 (average: 0.342) and 0.481 to 0.583 (average: 0.535) respectively, which are lower than the values previously obtained for *Paeonia decomposita* [48], *Magnolia sinostellata* [49], and *Camellia nitidissima* [50], thus indicating that the genetic diversity of *M. savatieri* is lower than that of these three species. For plants, reproductive patterns, genetic drift, natural selection, anthropogenic activities, and habitat fragmentation are among the main factors contributing to changes in genetic diversity [51]. It can be speculated that the low genetic diversity of *M. savatieri* populations is attributable to its narrow range of distribution, along with the large spatial distance between populations, which limits inter-population gene exchange. Moreover, excessive harvesting of natural populations, habitat destruction, and declining population sizes may contribute to inbreeding and genetic drift within the *M. savatieri* population, thereby reducing its genetic diversity. Nevertheless, among the four regional populations examined in this study, we identified the Zhejiang population as being the genetically most diverse. Generally, a higher genetic diversity is indicative of a higher complexity of plant diversity, and consequently a greater potential for environmental adaptation [44]. Accordingly, analysis of the genetic diversity of *M. savatieri* will predictably provide a theoretical basis for its protection and breeding.

The degree of differentiation between natural populations can be described in terms of gene flow (Nm) and the genetic differentiation coefficient (Fst), which are negatively correlated, with a higher differentiation coefficient between populations being taken to be indicative of a lower extent of gene flow [52]. Genetic differentiation among plant populations is influenced by range size, habitat fragmentation, and population isolation, reflecting the cumulative effects of genetic mutation, genetic drift, gene flow, and natural selection [53]. Previously, Wright et al. [54] established that genetic differentiation among populations can be considered high when Fst values are greater than 0.25, and thus the Fst value of 0.497 we obtained for *M. savatieri* would tend to indicate a significant genetic differentiation among populations. This assumption was supported by our AMOVA results (*P* < 0.001), indicating a higher molecular variation among populations (49.74%) than within populations (48.36%). Gene flow is one of the main factors contributing to the maintenance of a homogeneous population genetic structure. Theoretically, at Nm values > 1, there is a lower likelihood of genetic drift, thereby limiting the genetic differentiation among populations, whereas conversely, when Nm < 1, gene flow is insufficient to counteract the effects of genetic drift, thereby favoring an increase in the genetic differentiation among populations [55]. In the present study, we obtained a value of only 0.253 for the gene flow between *M. savatieri* populations, indicating that low levels of gene exchange would not weaken the differentiation among populations caused by genetic drift, which we suspect could be the primary factor influencing the genetic structure of *M. savatieri*. It can be reasoned that these findings reflect the characteristics of *M. savatieri*, such as its semi-parasitism, low reproductive capacity, and low seed viability. Moreover, the viability of *M. savatieri* populations is increasingly being threatened by habitat fragmentation and destruction, and consequently, declining numbers and hindered gene exchange may lead to a gradual increase in genetic differentiation.

The genetic structure of species reflects the mutation, recombination, genetic drift, and selection effects experienced by populations during the course of evolution [56], and thus population genetic structure analyses are deemed important for gaining an accurate understanding of the genetic relationships among germplasms. In this study, we used three clustering methods (PCoA, STRUCTURE, and UPGMA) to analyze the population structure of *M. savatieri*. PCoA and UPGMA clustering analyses were performed to classify the assessed materials based on their genetic similarity coefficients and genetic distance, which can clarify the intuitive relationship between populations [57]. However, the results obtained using these two clustering do not provide an indication of the materials that interpenetrated between populations. Conversely, Structure analysis is based on the Hardy-Weinberg equilibrium and Bayesian model algorithm, which eliminates the effects of deviation attributable human factors on population division and can objectively classify populations [58]. Consequently, in the present study, we used these three methods in combination to analyze germplasm resources, thereby enabling us to further characterize the germplasm population structure.

UPGMA clustering analysis divided the 46 individuals collected from 10 different wild regions into two clusters, thereby indicating the presence of two discrete genetic populations among these regions. Using STRUCTURE analysis, these individuals were clearly divided into three different clusters, with a few examples of admixed individuals. These results indicate the relatively homogeneous genetic structure of most samples, and the similar composition of samples collected from the same regions. The results of PCoA analysis were found to be identical to those obtained using STRUCTURE and were in general consistent with the UPGMA clustering tree. In addition, we found that the genetic relationships among populations reflected the natural geographical locations of these populations, which was supported by the Mantel test results, which revealed a significant positive correlation between the geographical and genetic distances among populations (*R*^2^ = 0.3304, *P* < 0.05). Collectively, these results tend to imply that geographical isolation, which would hinder gene exchange between different populations and isolate different gene pools, could be the primary factor contributing to the apparent genetic differentiation among *M. savatieri* populations.

## Conclusions

In the present study, 46 *M. savatieri* wild individuals were collected from 10 habitats in major distribution provinces in China. Using full-length transcriptome sequencing data, we identified and characterized EST-SSR loci and developed the first set of highly polymorphic EST-SSR markers for *M. savatieri*. The results of this study indicated that gene penetration among the different *M. savatieri* populations was relatively low, and the individuals from Zhejiang Province showed the highest genetic diversity. Geographical isolation plays a vital role in genetic differentiation among *M. savatieri* populations. Based on current trends, effective protection is absolutely necessary and urgent for wild germplasm resources of *M. savatieri* to maintain their genetic identity and diversity.

## Materials and methods

### Plant materials and DNA extraction

**Fig. 7:**
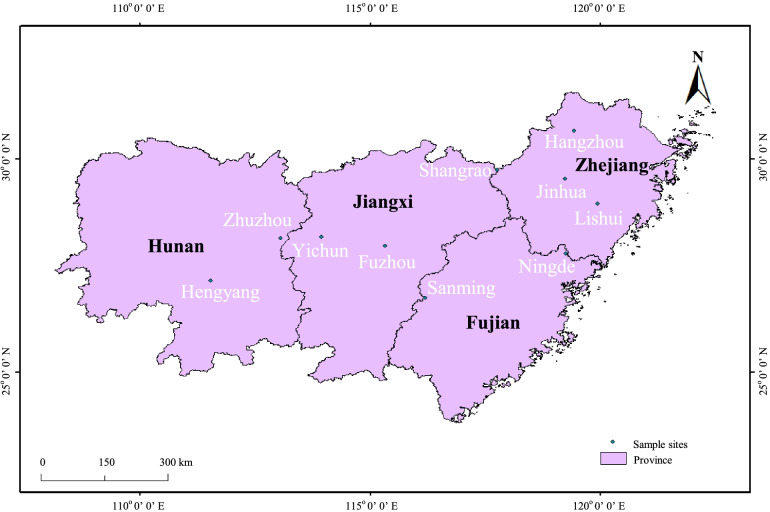
The sampling points of the wild *Monochasma savatieri* germplasm resources used in this study

For the purposes of the present study, we collected a total of 46 *M. savatieri* individuals from different distribution areas in China, among which there were nine accessions from Fujian Province (FJ), 11 from Hunan Province (HN), eight from Jiangxi Province (JX), and 18 from Zhejiang Province (ZJ) (Fig. 7, Supplemental Fig. 2). The formal identification of these plant materials was performed by Prof. Jiankun Xie (College of Life Sciences, Jiangxi Normal University, China). All plant materials collected in this study are conserved in the greenhouse at Jiangxi Normal University, China, and the seeds are freely available for scientific research. Information pertaining to these plants, including their source of origin, is presented in Supplemental Table 1. Fresh young leaves were collected and stored at − 80 °C for subsequent DNA extraction. Total genomic DNA was extracted from the frozen leaves using a Plant Genomic DNA Rapid Extraction Kit (Biotech, Shanghai, China), following the manufacturer’s recommendations. The DNA thus isolated was stored at − 20 °C for polymerase chain reaction (PCR) amplification.

### EST-SSRs identification and primers development

Full-length transcriptome sequencing data was obtained for the leaves and stems of *M. savatieri* by Biomarker Technologies (Beijing, China). The MISA (Microsatellite identification tool, http://pgrc.ipk-gatersleben.de/misa/) software was used to identify SSR loci from the transcript sequences (length ≥ 1000 bp) as previous described [59]. SSR search criteria was conducted based on momo-, di-, tri-, tetra-, penta-, and hexa-nucleotide motifs minimum number of 10, 6, 5, 5, 5, and 5 repeats, respectively. On the basis of these data, we randomly selected 50 EST-SSRs, for which we developed and synthesized the corresponding primer pairs. The selection parameters were as follows: a primer length of 18–25 bp, a GC content of 40–60%, and a melting temperatures of 55–65 °C. Additionally, the sequencing data have been deposited to the China National GeneBank (CNGB) with project accession number CNP0003034 (https://db.cngb.org/search/project/CNP0003034/).

### PCR amplification and polyacrylamide gel electrophoresis

For PCR amplification, we used 10 μL reaction mixtures, containing 1 μL (100 ng/μL) of genomic DNA, 5 μL of 2× FastTaq Premix (Tolo Biotech. Shanghai, China), 1 μL (0.01 nmol/μL) of the primers, and 3 μL of ddH_2_O. The amplification reaction program was as follows: pre-denaturation at 95 °C for 5 min; followed by 30 cycles of denaturation at 95 °C for 30 s, annealing at 55 °C for 45 s, and extension at 72 °C for 30 s; and a final extension at 72 °C for 10 min. The PCR products were detected using 8% denaturing polyacrylamide gels with 0.5× TBE buffer [60], run at 230 V for 80–90 min. The amplified products were visualized by silver staining the gels [61]. The patterns of bands amplified using the different SSR marker were analyzed on a gel imaging analysis system (version Tocan 240) (Shanghai Tocan Bio-Technology Co., Ltd.). We used Power Marker version 3.25 with default parameter settings [62] to compute the major allele frequency (MAF), gene diversity (GD), and polymorphic information content (PIC). In addition, the number of different alleles (Na), effective number of alleles (Ne), Shannon’s information index (I), expected heterozygosity (He), unbiased expected heterozygosity (uHe), percentage of polymorphic loci (PPL), fixation index (F), analysis of molecular variance (AMOVA), and principal coordinate analysis (PCoA) were computed using GenAlEx 6.5 software with default parameter settings [63]. Population structure was estimated using the Bayesian clustering method in STRUCTURE version 2.3.4 [64]. The length of the burn-in period and Markov Chain Monte Carlo (MCMC) run parameters were set to 100,000 iterations. We assessed K values of 1 to 10 based on 10 independent runs for each value. The most favorable K value was determined by estimating the maximum value of the ΔK statistic, using the web-based STRUCTURE Harvester (version 0.9.94) [65], and cluster analysis between populations, based on unweighted pair group with arithmetic average (UPGMA), was performed using NTSYS-pc (version 2.10e) software [66].

## Supplementary Information

**Additional file 1: Supplemental Table 1.** Details and sources of the 46 *Monochasma savatieri* samples analyzed in this study. **Supplemental Fig. 1.** The representative gel pictures with scoring by the EST-SSR markers. The patterns of bands were analyzed by a gel imaging analysis system (version Tocan 240). **Supplemental Fig. 2.** The photos of some representative samples in this study.

## Data Availability

All data generated or analysed during this study are included in this article. The sequencing data have been deposited to the CNGB with project accession number CNP0003034 (https://db.cngb.org/search/project/CNP0003034/). The transcript sequences used to detect EST-SSR loci of *Monochasma savatieri* were deposited and are available at https://github.com/AWan222/LRCyc.
