# Supplementary material for: Analysis of the genetic diversity and population structure of Monochasma savatieri Franch. ex Maxim using novel EST-SSR markers
Source: BMC Genomics. 2022 Aug 16;23:597. doi: 10.1186/s12864-022-08832-x (PMC9382759; doi:10.1186/s12864-022-08832-x)

Supplemental Table 1. Details and sources of the 46 *Monochasma savatieri* samples analyzed in this study

Population	Number	Voucher No.	Location	Longitude	Latitude
Fujian (FJ)	1	FJ-ND1	Ningde, Fujian	119.648	27.465
	2	FJ-ND2	Ningde, Fujian	119.648	27.465
	3	FJ-ND3	Ningde, Fujian	119.648	27.465
	4	FJ-ND4	Ningde, Fujian	119.648	27.465
	5	FJ-ND5	Ningde, Fujian	119.648	27.465
	6	FJ-SM1	Sanming , Fujian	116.664	26.451
	7	FJ-SM2	Sanming , Fujian	116.664	26.451
	8	FJ-SM3	Sanming , Fujian	116.664	26.451
	9	FJ-SM4	Sanming , Fujian	116.664	26.451
Hunan (HN)	10	HN-HY1	Hengyang , Hunan	112.104	26.845
	11	HN-HY2	Hengyang , Hunan	112.104	26.845
	12	HN-HY3	Hengyang , Hunan	112.104	26.845
	13	HN-HY4	Hengyang , Hunan	112.104	26.845
	14	HN-HY5	Hengyang , Hunan	112.104	26.845
	15	HN-HY6	Hengyang , Hunan	112.104	26.845
	16	HN-ZZ1	Zhuzhou, Hunan	113.585	27.815
	17	HN-ZZ2	Zhuzhou, Hunan	113.585	27.815
	18	HN-ZZ3	Zhuzhou, Hunan	113.585	27.815
	19	HN-ZZ4	Zhuzhou, Hunan	113.585	27.815
	20	HN-ZZ5	Zhuzhou, Hunan	113.585	27.815
Jiangxi (JX)	21	JX-FZ1	Fuzhou , Jiangxi	115.813	27.636
	22	JX-FZ2	Fuzhou , Jiangxi	115.813	27.636
	23	JX-FZ3	Fuzhou , Jiangxi	115.813	27.636
	24	JX-SR1	Shangrao , Jiangxi	118.198	29.383
	25	JX-SR2	Shangrao , Jiangxi	118.198	29.383
	26	JX-SR3	Shangrao , Jiangxi	118.198	29.383
	27	JX-SR4	Shangrao , Jiangxi	118.198	29.383
	28	JX-YC1	Yichun , Jiangxi	114.451	27.845
Zhejiang (ZJ)	29	ZJ-HZ1	Hangzhou , Zhejiang	119.832	30.281
	30	ZJ-HZ2	Hangzhou , Zhejiang	119.832	30.281
	31	ZJ-HZ3	Hangzhou , Zhejiang	119.832	30.281
	32	ZJ-HZ4	Hangzhou , Zhejiang	119.832	30.281
	33	ZJ-HZ5	Hangzhou , Zhejiang	119.832	30.281
	34	ZJ-HZ6	Hangzhou , Zhejiang	119.832	30.281
	35	ZJ-JH1	Jinhua , Zhejiang	119.636	29.185
	36	ZJ-JH2	Jinhua , Zhejiang	119.636	29.185
	37	ZJ-JH3	Jinhua , Zhejiang	119.636	29.185
	38	ZJ-JH4	Jinhua , Zhejiang	119.636	29.185
	39	ZJ-JH5	Jinhua , Zhejiang	119.636	29.185
	40	ZJ-JH6	Jinhua , Zhejiang	119.636	29.185

41	ZJ-LS1	Lishui , Zhejiang	120.328	28.613
42	ZJ-LS2	Lishui , Zhejiang	120.328	28.613
43	ZJ-LS3	Lishui , Zhejiang	120.328	28.613
44	ZJ-LS4	Lishui , Zhejiang	120.328	28.613
45	ZJ-LS5	Lishui , Zhejiang	120.328	28.613
46	ZJ-LS6	Lishui , Zhejiang	120.328	28.613

Supplemental Fig. 1 The representative gel pictures with scoring by the EST-SSR markers. The patterns of bands were analyzed by a gel imaging analysis system (version Tocan 240)

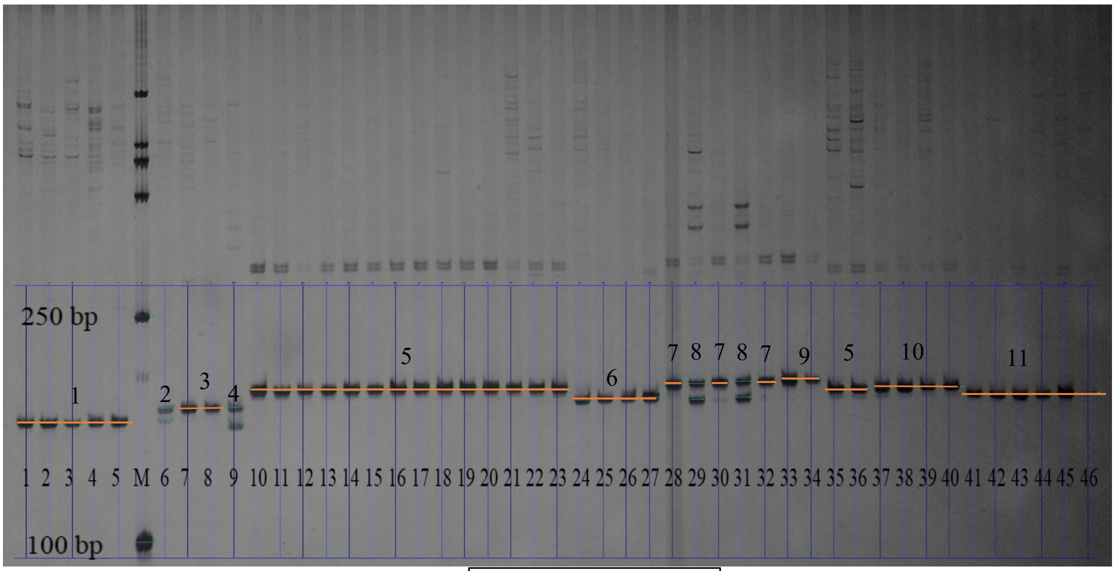

LRC-19013-3

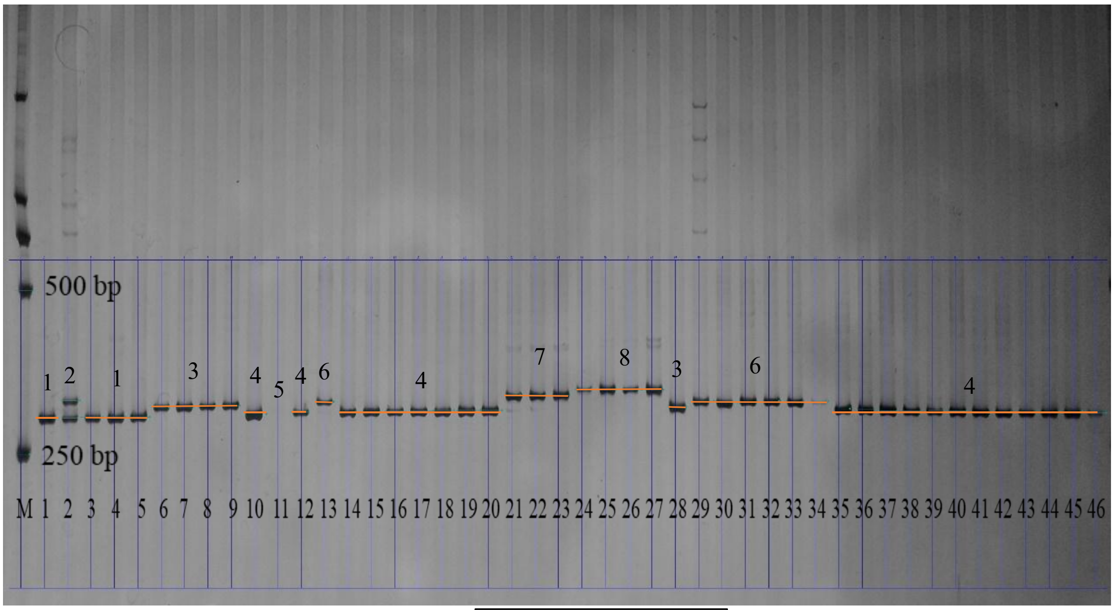

LRC-35320-1

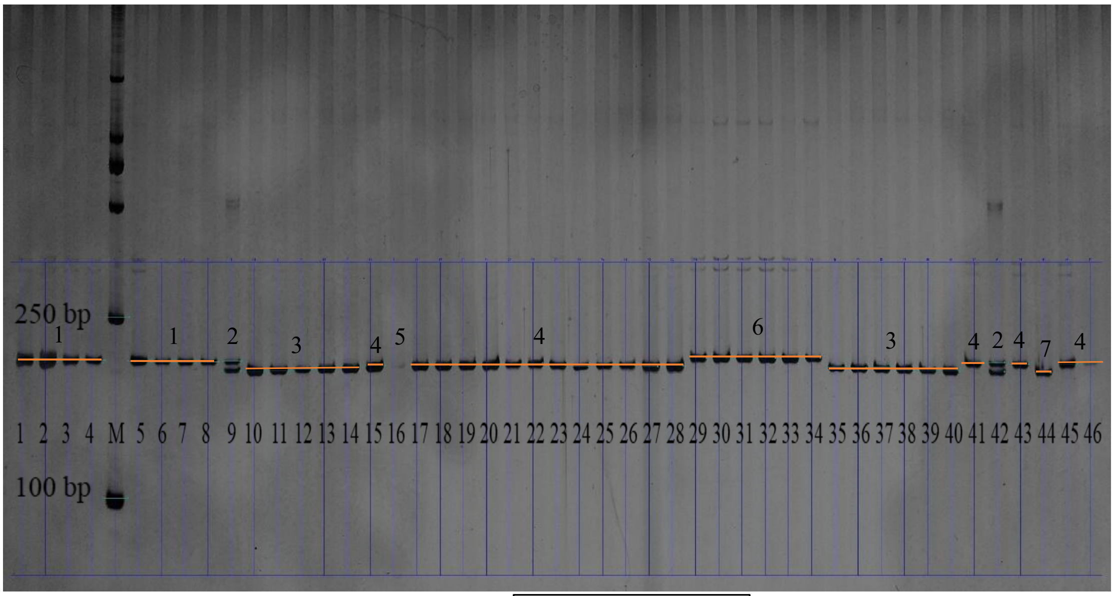
250 bp
100 bp
1 1 2 3 4 5 4 6 3 4 2 4 7 4
1 2 3 4 M 5 6 7 8 9 10 11 12 13 14 15 16 17 18 19 20 21 22 23 24 25 26 27 28 29 30 31 32 33 34 35 36 37 38 39 40 41 42 43 44 45 46

LRC-10353-1

Supplemental Fig. 2 The photos of some representative samples in this study

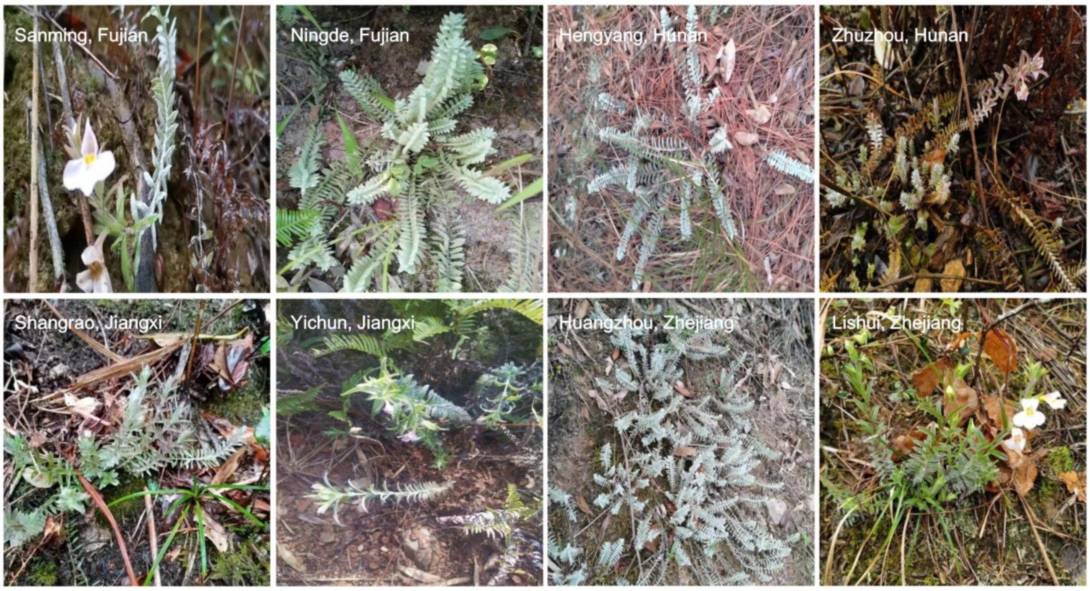

Supplement: Supplementary file 1 — Additional file 1: Supplemental Table 1. Details and sources of the 46 Monochasma savatieri samples analyzed in this study. Supplemental Fig. 1. The representative gel pictures with scoring by the EST-SSR markers. The patterns of bands were analyzed by a gel imaging analysis system (version Tocan 240). Supplemental Fig. 2. The photos of some representative samples in this study. [file 12864_2022_8832_MOESM1_ESM.pdf]
